# Textural, Structural and Biological Evaluation of Hydroxyapatite Doped with Zinc at Low Concentrations

**DOI:** 10.3390/ma10030229

**Published:** 2017-02-25

**Authors:** Daniela Predoi, Simona Liliana Iconaru, Aurélien Deniaud, Mireille Chevallet, Isabelle Michaud-Soret, Nicolas Buton, Alina Mihaela Prodan

**Affiliations:** 1 National Institute of Materials Physics, P.O. Box MG 07, 07725 Magurele, Romania; simonaiconaru@gmail.com; 2 CNRS, Laboratoire de Chimie et Biologie des Métaux (LCBM) UMR 5249 CNRS-CEA-UJF, F-38054 Grenoble, France; aurelien.deniaud@cea.fr (A.D.); mireille.chevallet@cea.fr (M.C.); isabelle.michaud-soret@cea.fr (I.M.-S.); 3 CEA, LCBM, F-38054 Grenoble, France; 4 LCBM, University Grenoble Alpes, LCBM, F-38054 Grenoble, France; 5 HORIBA Jobin Yvon S.A.S., 6-18, rue du Canal, 91165 Longjumeau Cédex, France; nicolas.buton@horiba.com; 6 Emergency Hospital Floreasca Bucharest, 8 Calea Floresca, Sector 1, 014461 Bucharest, Romania; prodan1084@gmail.com; 7 Surgery Department, Carol Davila University of Medicine and Pharmacy, 8 Eroii Sanitari, Sector 5, 050474 Bucharest, Romania

**Keywords:** zinc, hydroxyapatite, *Staphylococcus aureus*, *Escherichia coli*, HepG2 cell viability

## Abstract

The present work was focused on the synthesis and characterization of hydroxyapatite doped with low concentrations of zinc (Zn:HAp) (0.01 < *x*_Zn_ < 0.05). The incorporation of low concentrations of Zn^2+^ ions in the hydroxyapatite (HAp) structure was achieved by co-precipitation method. The physico-chemical properties of the samples were characterized by X-ray Diffraction (XRD), Fourier Transform Infrared Spectroscopy (FTIR), X-ray photoelectron spectroscopy (XPS), Scanning Electron Microscopy (SEM), zeta-potential, and DLS and N_2_-BET measurements. The results obtained by XRD and FTIR studies demonstrated that doping hydroxyapatite with low concentrations of zinc leads to the formation of a hexagonal structure with lattice parameters characteristic to hydroxyapatite. The XRD studies have also shown that the crystallite size and lattice parameters of the unit cell depend on the substitutions of Ca^2+^ with Zn^2+^ in the apatitic structure. Moreover, the FTIR analysis revealed that the water content increases with the increase of zinc concentration. Furthermore, the Energy Dispersive X-ray Analysis (EDAX) and XPS analyses showed that the elements Ca, P, O, and Zn were found in all the Zn:HAp samples suggesting that the synthesized materials were zinc doped hydroxyapatite, Ca_10−*x*_Zn*_x_*(PO_4_)_6_(OH), with 0.01 ≤ *x*_Zn_ ≤ 0.05. Antimicrobial assays on *Staphylococcus aureus* and *Escherichia coli* bacterial strains and HepG2 cell viability assay were carried out.

## 1. Introduction

In the last decades, researchers worldwide have focused their attention on the development of new and improved biomaterials for different biomedical applications [1,2,3,4]. One of the main concerns in the medical field is finding new ways to treat bone diseases such as osteoporosis. According to the International Osteoporosis Foundation, over 70 million people from Europe, USA and Japan are affected by this disease. Due to the fact that in most cases the installation of osteoporosis does not exhibit any symptoms, one of the first indication of this disease being a fractured bone, physicians from all around the world are trying to create synthetic materials that can not only mimic the human hard tissue, but also improve the quality of the host tissue. Considering that hydroxyapatite (HAp), having the chemical formula Ca_10_(PO_4_)_6_(OH)_2_, is the main inorganic constituent of human bones, synthetic HAp is already used in bone graft surgeries [1,2,3,4]. Hydroxyapatite is a biomaterial used on a large scale due to its outstanding biocompatible and bioactive properties [5].

Previous studies [5,6] have demonstrated that, when implanted, HAp has the ability to create bonds with the surrounding bone, thus promoting bone formation, which is a very important step in the process of the implant osteointegration [5,6]. However, there are cases where the physico-chemical properties of hydroxyapatite are not sufficient. Therefore, in order to enhance the properties of HAp, substitutions with different ions found in the natural hard tissue could be achieved. In the structure of natural apatites can be found different metal ions such as Mg^2+^, Sr^2+^, Zn^2+^ or Mn^2+^. Among these ions, zinc is one of the most important trace elements, being found in human bones and plasma [7,8,9]. Zinc is also the second most frequently found transition metal in the human body after iron, being responsible for proper human growth and development [10,11,12,13,14]. In the natural bone tissue, can be found high quantities of zinc and its role is to preserve bone mineral density and bone metabolism [10]. Considering that every stage of the bone metabolism requires different amounts of zinc, a decrease of the optimum amount of this transition element leads to the onset of osteoporosis [10].

In order to improve the quality of hydroxyapatite based bone implants by adding the properties of different metal ions already present in the natural surrounding tissue, researchers have concluded that one of the best methods to preserve and enhance the existing properties is to dope the hydroxyapatite [10,11,12,13,14,15,16,17]. Therefore, taking into account the specific properties of zinc and its role in the proper functioning of the human body, it is easy to understand why much attention has been paid to the integration of Zn^2+^ ions in the structure of hydroxyapatite. The combination of HAp and Zn alike might enable the possibility of creating a new biomaterial which could improve the treatment for osteoporosis on the one hand and its prevention on the other hand.

The present study is focused on the synthesis of zinc doped hydroxyapatite (Zn:HAp, Ca_10−*x*_Zn*_x_*(PO_4_)_6_(OH)_2_) with crystal phase assigned to the hexagonal Ca_10_(PO_4_)_6_(OH)_2_ in P6_3/m_ space group. The nature of the crystal phase was determined by powder X-ray diffraction (XRD). Scanning Electron Microscopy (SEM) and Fourier transform infrared (FTIR) spectroscopy were also used to investigate the structural and morphological properties of zinc doped hydroxyapatite (Zn:HAp). XPS measurements were also performed. Furthermore, cytotoxicity assays were performed on two prokaryotic models, *Staphylococcus aureus* and *Escherichia coli*, as well as one eukaryotic model, HepG2 hepatocytes.

## 2. Results and Discussion

**Figure 1:**
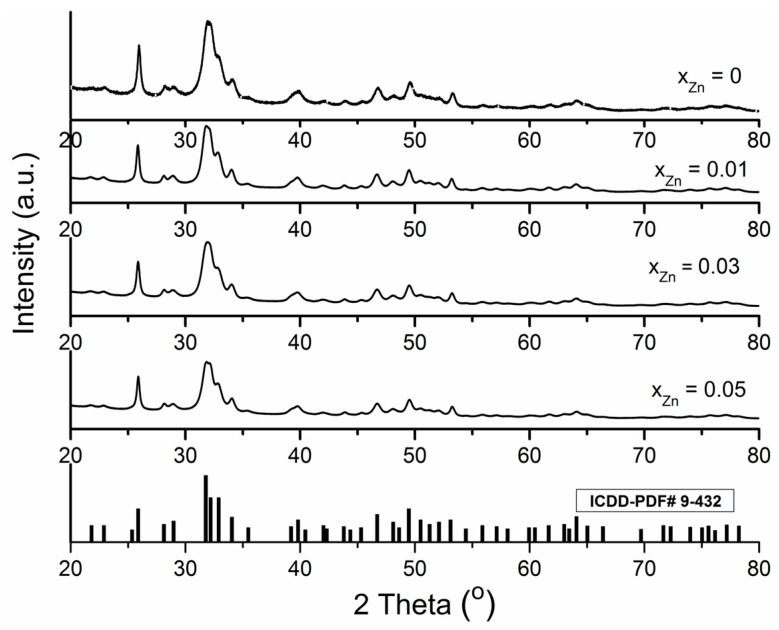
The XRD (X-ray diffraction) patterns of HAp and Zn:HAp, Ca_10−*x*_Zn*_x_*(PO_4_)_6_(OH)_2_ synthesized samples with 0 ≤ *x*_Zn_ ≤ 0.05.

The XRD patterns of the zinc doped hydroxyapatite powders with *x*_Zn_ = 0.01, *x*_Zn_ = 0.03 and *x*_Zn_ = 0.05 are shown in Figure 1. For the present study, the amount of zinc was chosen to be 0, 1, 3, 5 at %, with respect to Zn/(Zn+Ca) ratio. X-ray diffraction results of all Zn:HAp samples (*x*_Zn_ = 0.01, *x*_Zn_ = 0.03 and *x*_Zn_ = 0.05) show only the peaks assigned to the hexagonal Ca_10_(PO_4_)_6_(OH)_2_ in P6_3/m_ space group, according to the standard ICDD-PDF No. 9-432.

Figure 1 exhibits the Rietveld refinement of X-ray diffraction data for the Zn:HAp samples, with *x*_Zn_ = 0.0, *x*_Zn_ = 0.01, *x*_Zn_ = 0.03 and *x*_Zn_ = 0.05. The diffractograms of Zn:HAp samples are similar and the diffraction peaks become broader when the zinc concentration from the samples increases. According to previous studies conducted by Miyaji et al. [18], this behavior suggests that the crystallinity of the apatite significantly decreases with the increase of zinc concentration.

In all the diffractograms of Zn:HAp samples with various zinc concentrations only a hexagonal phase with lattice parameters that are characteristic to hydroxyapatite was indentified (Table 1). The average crystallite sizes measured parallel (DII) and perpendicular (D⊥) to the c axis decreased with the increase of zinc concentration (Table 1). The lattice parameter “a” increased when the zinc concentration in the samples increased from *x*_Zn_ = 0 to *x*_Zn_ = 0.05. On the other hand, the lattice parameter “c” decreased with the increase of zinc concentration in the samples from *x*_Zn_ = 0 to *x*_Zn_ = 0.05. A similar behavior was observed by Bigi et al. [19] in previous studies on the inhibition effect of zinc on hydroxyapatite crystallization. Moreover, these authors [19] have shown that the values of lattice parameters “a” and “c” are influenced by the synthesis method and the pH at which the synthesis is carried out.

**Figure 2:**
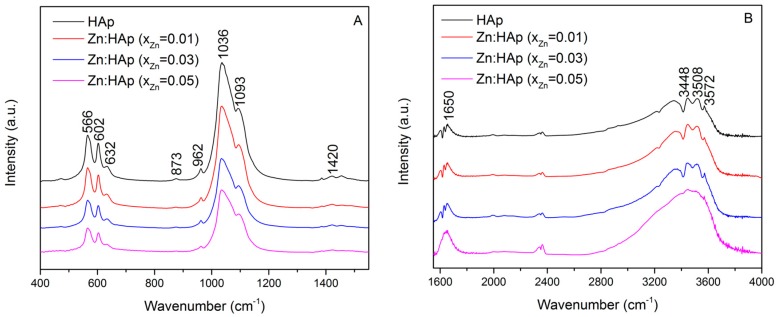
Fourier transform infrared spectroscopy (FTIR) spectra of Zn:HAp, samples with 0 ≤ *x*_Zn_ ≤ 0.05 from 400 to 1400 cm^−1^ (**A**) and from 1600 to 4000 cm^−1^ (**B**).

The composition of the prepared samples has been investigated through FTIR measurements. The spectra obtained for the hydroxyapatite doped with low concentrations of zinc are presented in Figure 2A,B. The content of the studied samples is comprised of several functional groups. All the bands are assigned to different vibrational modes of phosphate, carbonate and hydroxyl groups. Figure 2B exhibits the region consisting of vibrational bands associated with the presence of water lattice.

The bands registered at 566 cm^−1^ and 602 cm^−1^ correspond to the bending mode of the phosphate group [20,21,22,23,24]. Furthermore, the presence of functional groups in the prepared samples is also highlighted by the bands found at approximately 960 cm^−1^ [25,26] and 1093 cm^−1^ [25,27]. The vibrational bands found in the spectral regions 870–880 cm^−1^ and 1036–1050 cm^−1^ correspond to carbonate and phosphate groups, although it is difficult to separate them. At around 873 cm^−1^, a vibrational band characteristic to the phosphate group can be found [2], which overlaps with the 875 cm^−1^ band characteristic to the carbonate group [28]. The same behavior occurs at around 1420 cm^−1^. The vibrational bands of ${\mathrm{PO}}_{4}^{3-}$ present in the spectral region 1036–1042 cm^−1^ [1] are intertwined with the bands associated to the carbonate groups present in the apatite structure found in the region 1420–1450 cm^−1^ [28]. The presence of the hydroxyl group is evidenced by the bands observed at 3572 cm^−1^ and 3448 cm^−1^ assigned to the stretching mode [29,30,31], the band from 1650 cm^−1^ which corresponds to the deformation of O–H bond [32] and the band from around 632 cm^−1^ associated with the hydroxyl librational mode (L(OH)) [32,33]. The band found at 3508 cm^−1^ is caused by the adsorbed water present in the samples. Moreover, the FTIR spectra of hydroxyapatite doped with low concentrations of zinc showed that the chemical composition of all the samples was HAp. In addition, the separation of ${\mathrm{PO}}_{4}^{3-}$ stretching peaks at 960 and 1036 cm^−1^ is not clear when the concentration of zinc increases. These changes in the FTIR spectra suggest that the crystallinity of the apatite decreased with the increase of zinc concentration. These results are in good agreement with the XRD results. A similar behavior was also observed by Bigi et al. in previous studies [19]. On the other hand, it can be observed (Figure 2B) that the water content increases with the increase of zinc concentration. The vibrational bands associated with the water lattice tend to be more intense when the concentration of zinc increases from *x*_Zn_ = 0.01 to *x*_Zn_ = 0.03.

**Figure 3:**
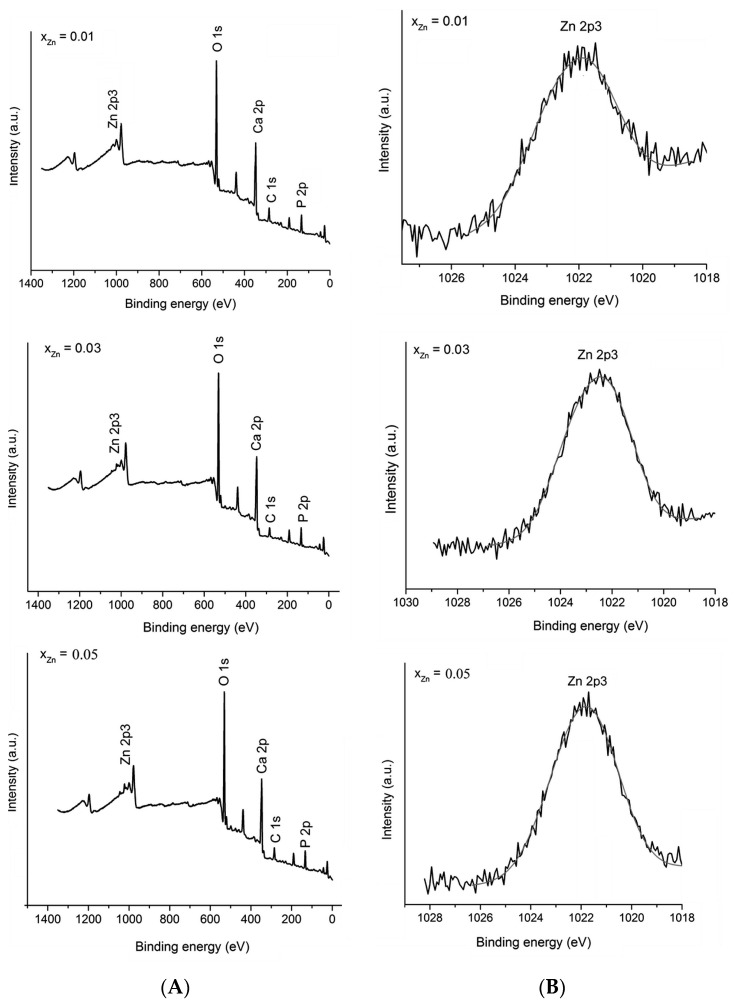
XPS general spectrum of Zn:Hap, Ca_10−*x*_Zn*_x_*(PO_4_)_6_(OH)_2_ samples synthesized with 0.01 ≤ *x*_Zn_ ≤ 0.05 (**A**); and narrow scan spectra of Zn element (**B**).

X-ray photoelectron spectroscopy (XPS) measurements were performed in order to investigate the successful doping of HAp with Zn (Figure 3). High resolution spectra of the Zn 2p3 regions were obtained. Figure 3A presents the XPS general spectrum of Ca_10−*x*_Zn*_x_*(PO_4_)_6_(OH)_2_ with different concentration of zinc (0.01 ≤ *x*_Zn_ ≤ 0.05). In the XPS general spectrum it can easily be observed the presence of calcium (Ca 2p), phosphorus (P 2p) and oxygen (O 1s) in the studied samples. On the other hand, the major peak assigned to Zn 2p3 was observed at a binding energy value of around 1022 eV. Figure 3B shows the high resolution XPS scans of the Zn 2p3 corresponding to Zn:HAp samples with 0.01 ≤ *x*_Zn_ ≤ 0.05. The (Ca+Zn)/P molar ratios determined from XPS was 1.62 ± 0.2 for the samples with 0.00 ≤ *x*_Zn_ ≤ 0.03. The XPS spectra provide us structural information about the Zn:HAp samples and suggest that the synthesized materials were zinc doped hydroxyapatite (Ca_10−*x*_Zn*_x_*(PO_4_)_6_(OH)_2_). The peaks assigned to Zn 2p3 were in agreement with those reported by Moulder et al. [34]. The results of the physico-chemical analysis clearly demonstrate that the Zn^2+^ ions replaced the Ca^2+^ ions and were structurally incorporated into the HAp crystal structure. 

**Figure 4:**
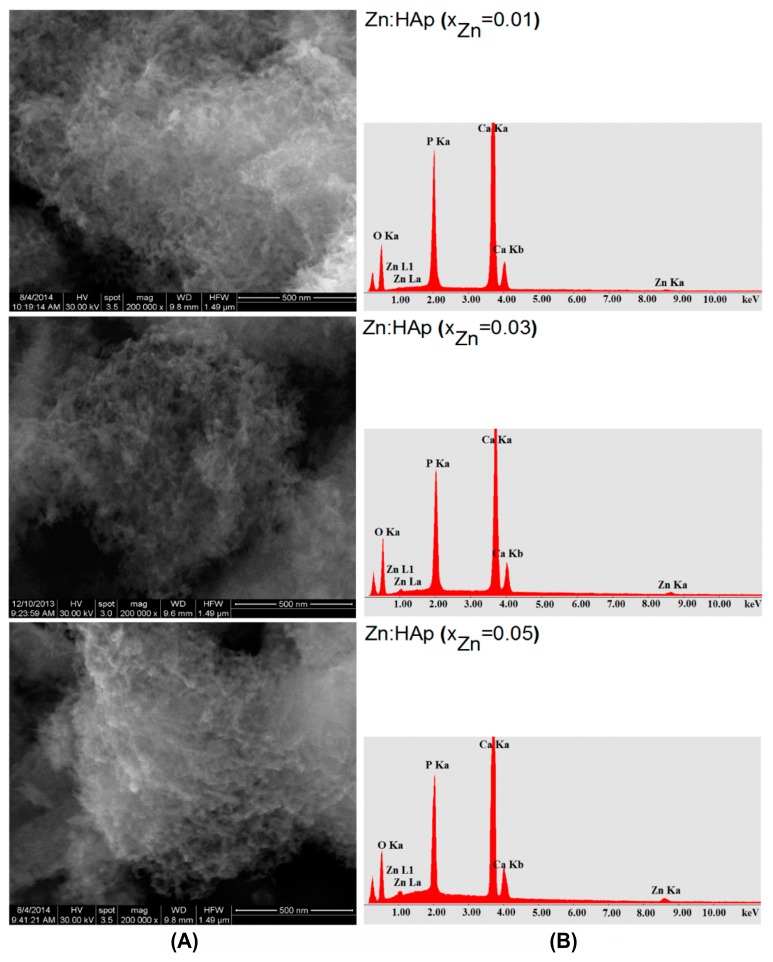
Scanning electron microscopy (SEM) images (**A**); and the EDAX analysis (**B**) of Zn:HAp, samples with 0.01 ≤ *x*_Zn_ ≤ 0.05.

SEM images and EDAX analysis for the Zn:HAp (0.01 ≤ *x*_Zn_ ≤ 0.05) samples obtained by adapted precipitation method are shown in Figure 4A,B. SEM images (Figure 4A) show that the particle size decreases when the zinc concentration increases. The morphology of Zn:HAp samples was similar for all zinc concentration and shape of the particles remained unchanged. Furthermore, SEM images have revealed an agglomeration of the particles analyzed. On the other hand, a slight decrease in particle size was observed when the zinc concentration increases. SEM analysis and XRD studies results were consistent with previous studies on physicochemical and biological properties of iron and zinc ions co-doped nanocrystalline hydroxyapatite, synthesized by ultrasonication conducted by Ramya et al. [35]. The elements of Ca, P, O, and Zn were found in all the Zn:HAp samples, Ca_10−*x*_Zn*_x_*(PO_4_)_6_(OH) (Figure 4B). The mean molar ratio (Ca+Zn)/P was 1.66 ± 0.1 for the samples with 0.00 ≤ *x*_Zn_ ≤ 0.03. The value of (Ca+Zn)/P ratio found by EDAX analysis is close to the value of stoichiometric hydroxyapatite (1.67). 

**Figure 5:**
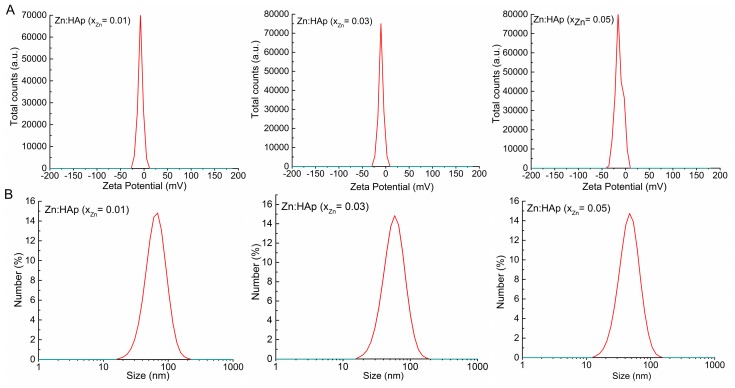
Colloidal characteristics of the Zn:Hap, Ca_10−*x*_Zn*_x_*(PO_4_)_6(_OH)_2_ samples synthesized with 0.01 ≤ *x*_Zn_ ≤ 0.05: (**A**) zeta-potential distribution curves; and (**B**) hydrodynamic size distribution curves images.

The colloidal characteristics of the Zn:HAp, Ca_10−*x*_Zn*_x_*(PO_4_)_6_(OH)_2_ samples synthesized with 0.01 ≤ *x*_Zn_ ≤ 0.05 are presented in Figure 5A,B.

The zeta potential of Ca_10−*x*_Zn*_x_*(PO_4_)_6_(OH)_2_ nanoparticles (0.01 ≤ *x*_Zn_ ≤ 0.05) was established from their electrophoretic mobility (U_E_) using Smoluchowski equation:

U_E_ = εξ/η (1)

where ε is the dielectric constant, ξ is zeta potential, and η is the viscosity of the medium. The zeta potential value of the Zn:HAp nanocomposites (Figure 5A) was −8.19 mV (*x*_Zn_ = 0.01), −10.95 mV (*x*_Zn_ = 0.03) and −15.84 mV (*x*_Zn_ = 0.05). The value of zeta potential of the Zn:HAp nanocomposites are in agreement with the previous studies conducted by Sanaa et al. [36]. In their studies on ceria-containing uncoated and coated hydroxyapatite-based galantamine nanocomposites for formidable treatment of Alzheimer’s disease in ovariectomized albino-rat model, M.R. Sanaa et al. showed that zeta potential was changed from −9 mV for pure hydroxyapatite to −20 and −15 mV for nanoceria containing hydroxyapatite based-galantamine nanocomposites. Moreover, the zeta potential value of the pure HAp obtained by Liu et al. [37] in the previous studies was almost zero (−5.20 mV).

The mean particle size of Zn:HAp samples synthesized by an adapted co-precipitation method under the controllable conditions after diluted in distilled water was determined by DLS analysis (Figure 5B). The mean particle size of Zn:HAp samples determined by DLS were evaluated to be 47 nm (*x*_Zn_ = 0.05), 58 (*x*_Zn_ = 0.03) and 67 (*x*_Zn_ = 0.01). A comparison between the DLS evaluation revealed that the aggregate sizes decreased when the concentration of zinc increased in the samples from *x*_Zn_ = 0.01 to *x*_Zn_ = 0.05. According to Keller et al. [38], this behavior play an important role in determining the toxicity because it is unlikely that the cells to be exposed to Zn:HAp nanoparticles in their original size. Besides, the size and size distribution of the particles can have a substantial influence on both the assimilation and biological destiny of particles [39]. Moreover, the DLS evaluation confirmed the results of SEM investigations.

**Figure 6:**
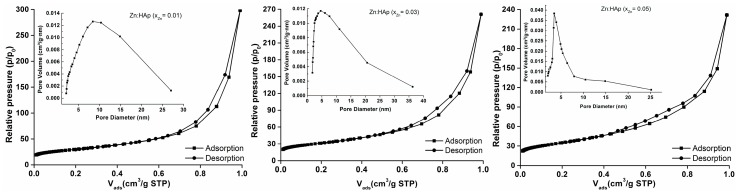
Textural characteristics of the Zn:HAp, Ca_10−*x*_Zn*_x_*(PO_4_)_6_(OH)_2_ samples synthesized with 0.01 ≤ *x*_Zn_ ≤ 0.05 using N_2_ adsorption–desorption isotherms and the pore size distribution curves.

The textural characteristics of the Zn:HAp, Ca_10−*x*_Zn*_x_*(PO_4_)_6_(OH)_2_ samples synthesized with 0.01 ≤ *x*_Zn_ ≤ 0.05 using N_2_ adsorption–desorption isotherms and the pore size distribution curves are presented in Figure 6.

The Brunauer–Emmett–Teller (BET) N_2_ adsorption/desorption analyses [40] were accomplished to determine the BET surface area (S_BET_), V_m_ (the volume of gas adsorbed at standard temperature and pressure (STP) (273.15 K and atmospheric pressure (1.013 × 10^5^ Pa)) to produce an apparent monolayer on the sample surface) of the samples. The values of V_m_ were calculated using the slope and intercept of a BET plot. The dimensionless constant that is related to the enthalpy of adsorption of the adsorbate gas on the powder sample, C, was also calculated. On the other hand, the Barrett–Joyner–Halenda (BJH) N_2_ adsorption/desorption analyses [41] were conducted to measure the pore size and pore volume of the Zn:HAp (0.01≤ *x*_Zn_ ≤ 0.05) samples. As can be seen, adsorption isotherms obtained are type II according to the classification BDDT [42]. Type II isotherms are characteristic materials showing a mixture of micro- and mesoporous. Moreover, according to DeBoer’s classification [43], the type of hysteresis observed shows that the form of the pores of samples analyzed is type A. According to DeBoer, the pores of type A are tubular pores. The reducing of hysteresis shows that some of the tubular pores are closed at one end. A summary of the isotherms results is presented in Table 2.

**Figure 7:**
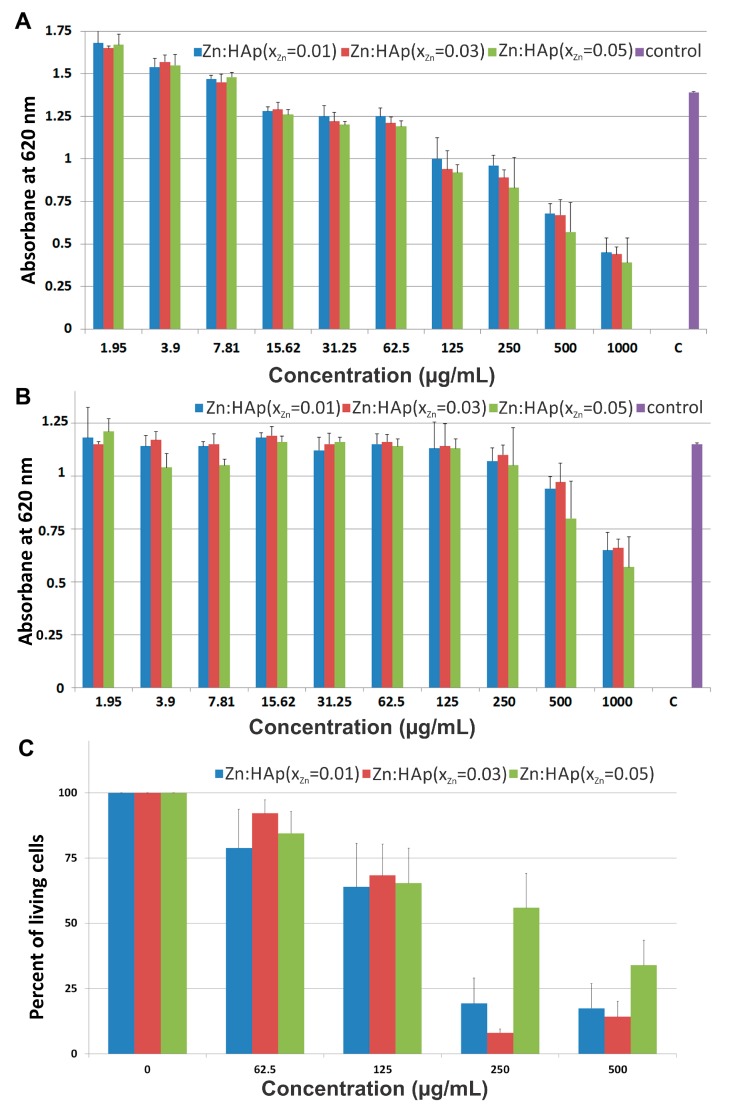
Cell viability assays: (**A**) *S. aureus* cell growth in LB at 30 °C for 12 h in the presence of Zn:HAp with *x*_Zn_ = 0.01, *x*_Zn_ = 0.03 or *x*_Zn_ = 0.05 at concentrations between 1.95 and 1000 µg/mL; (**B**) *E. coli* cell growth in LB at 30 °C for 12 h in the presence of Zn:HAp with *x*_Zn_ = 0.01, *x*_Zn_ = 0.03 or *x*_Zn_ = 0.05 at concentrations between 1.95 and 1000 µg/mL; and (**C**) HepG2 viability after a 24 h incubation with Zn:HAp with *x*_Zn_ = 0.01, *x*_Zn_ = 0.03 or *x*_Zn_ = 0.05 at concentrations between 62.5 and 500 µg/mL. Error bars are calculated from at least 3 independent experiments.

The effect of Zn:HAp with *x*_Zn_ = 0.01, *x*_Zn_ = 0.03 and *x*_Zn_ = 0.05 on cell viability was assessed on prokaryote and eukaryote cells (Figure 7). *Staphylococcus aureus* (*S. aureus*) and *Escherichia coli* (*E. coli*) were chosen to represent gram-positive and gram-negative bacteria, respectively. One of the objectives of this study was to obtain important information about the antibacterial effect of Zn:HAp on *S. aureus* and *E. coli*. These are common bacteria representing common infections which are facing food, pharmaceutical and medical industry.

The effect of the synthesized Zn:HAp, Ca_10−*x*_Zn*_x_*(PO_4_)_6_(OH)_2_ with 0.01 ≤ *x*_Zn_ ≤ 0.05 tested against *S. aureus* cell growth at various concentrations from 1.95 to 1000 µg/mL are presented in Figure 7A. *S. aureus* cell growth was diminished at concentrations greater than 125 µg/mL for the three Zn:HAp tested (Figure 7A). An impaired cell growth of *S aureus* was also observed at concentrations between 31.25 and 125 µg/mL for the three Zn:HAp tested. No effect on *S aureus* cells growth of ZnHAp was noticed at concentrations lower than 31.25 µg/mL.

Besides, the three different Zn:HAp showed an effect on *Escherichia coli* cell growth only at the very high concentrations (1000 and 500 µg/mL). Zn:HAp with *x*_Zn_ = 0.01 and *x*_Zn_ = 0.03 inhibit *E. coli* growth by 45% and 20% at 1000 and 500 µg/mL (Figure 7B), respectively. Zn:HAp with *x*_Zn_ = 0.05 inhibits growth even further, reaching 50% and 35% inhibition for the same concentrations (Figure 7B). At 250 µg/mL, we observed a slight effect for all three Zn:HAp compounds with a decrease in absorbance of only 10%. Lower concentrations showed no effect on *E. coli* growth.

In HepG2 cells, we tested cell viability in the presence of Zn:HAp with *x*_Zn_ = 0.01, *x*_Zn_ = 0.03 and *x*_Zn_ = 0.05 at four different concentrations of 62.5, 125, 250 and 500 µg/mL. At higher concentrations, in the absence of agitation, hydroxyapatite particles sedimented in a thick layer over the cells and caused massive cell death (see below). For the lower concentrations, we observed a similar effect for Zn:HAp with *x*_Zn_ = 0.01, *x*_Zn_ = 0.03 and *x*_Zn_ = 0.05 with a 20% lethal dose (LD_20_) around 62.5 µg/mL and a LD_50_ above 125 µg/mL (Figure 7C). Cell mortality at 250 and 500 µg/mL for both *x*_Zn_ = 0.01 and *x*_Zn_ = 0.03 was above 80%. Surprisingly, the Zn:HAp with *x*_Zn_ = 0.05 is less toxic: 55% and 35% of cells were viable at 250 and at 500 µg/mL, respectively. Toxicity observed with the different compounds is most probably due to sedimentation of the particles during the experiment. The sedimentation of the particle is a problem for the cell viability assay only at the highest concentration and does not hinder our conclusions concerning the biological effect of Zn-HAp. However, the positive effect on cell survival observed for Zn:HAp with *x*_Zn_ = 0.05 could be due to a higher release of Zn(II) ions that are known to stimulate cell proliferation at concentrations in the 100 µM range [44].

**Figure 8:**
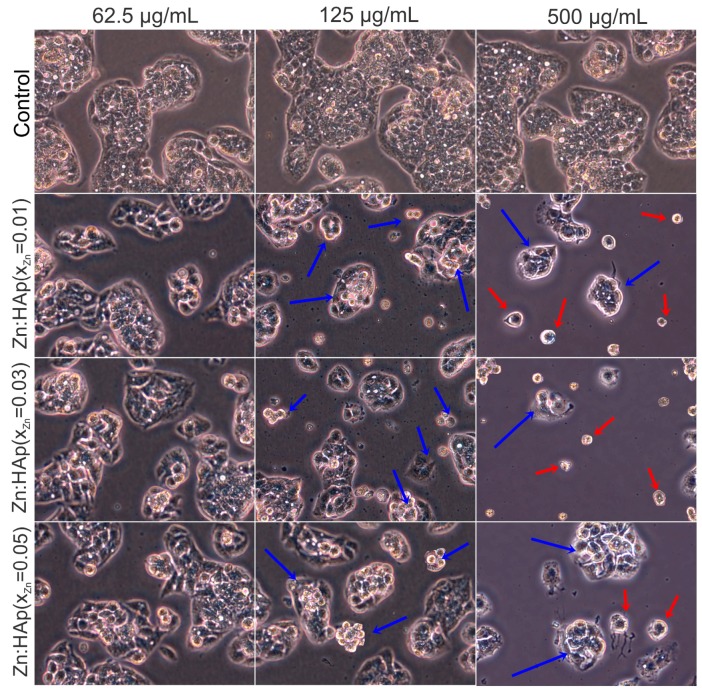
Inverted phase contrast microscopy of HepG2 cells cultivated in the presence of Zn:HAp with *x*_Zn_ = 0.01, *x*_Zn_ = 0.03 and *x*_Zn_ = 0.05 at three different concentrations (62.5, 125 and 500 µg/mL) compared to control.

Morphological changes in HepG2 cells after 24-h treatment with 62.5, 125 and 500 µg/mL Zn:HAp (*x*_Zn_ = 0.01, *x*_Zn_ = 0.03 and *x*_Zn_ = 0.05) are presented in Figure 8. According to Herzog et al. [45], during the development of drugs, primary human hepatocytes are widely used to identify drug metabolites and to investigate possible liver toxicity. Control cells were not treated with Zn:HAp. Control cells showed the normal morphology of HepG2 cells. The HepG2 cells treated with 62.5 µg/mL Zn:HAp (*x*_Zn_ = 0.01, *x*_Zn_ = 0.03 and *x*_Zn_ = 0.05) for 24 h did not show morphological changes. Moreover, the morphology of HepG2 cells treated with 125 µg/mL Zn:HAp (*x*_Zn_ = 0.05) for 24 h remained unchanged but apoptotic cells were present (blue arrows). The HepG2 cells treated with 125 µg/mL Zn:HAp (*x*_Zn_ = 0.01 and *x*_Zn_ = 0.03) presented apoptotic features (blue arrows) after 24 h. On the other hand, the morphology of HepG2 cells treated with 500 µg/mL (*x*_Zn_ = 0.01, *x*_Zn_ = 0.03 and *x*_Zn_ = 0.05) is changed. At these concentrations, apoptotic cells (blue arrows) and detached cells (red arrows) are observed [46].

In summary, a significant cytotoxicity was observed on *S. aureus* while a very low cytotoxicity was observed on *E. coli* bacteria and in the same range for the three differently doped HAp. The cytotoxic effects are stronger in the case of hepatic cells. Indeed, a decrease of the viability starts to appear clearly at 125 µg/mL. Interestingly, this cytotoxicity is significantly lower in higher Zn content. This may be due to a release of Zn ions or to a difference in solubility of the three Zn-HAp. Consequently, we suggest that the strategies that aim to use hydroxyapatite doped with different metal ions (such as Ag^+^, Cu^2+^, and Zn^2+^) for possible applications in medical or pharmaceutical field for their antimicrobial properties should take into account the adverse effects thereof.

The goal of this research was to study the effect of doping hydroxyapatite with zinc ions. The results above show the influence of Zn:HAp nanoparticles on *S. aureus* growth, which is in agreement with previous studies conducted by Tank et al. [47]. Previous studies on pure and zinc doped nano-hydroxyapatite conducted by Tank et al. showed that the zinc doped hydroxyapatite nanoparticles obtained by surfactant mediated chemical precipitation route shows a good antimicrobial activity against *Staphylococcous aureus* MTCC 1430. Moreover, Anwar et al. [48], in their previous studies, showed that the nanoscale zinc substituted hydroxyapatite bioceramics obtained by continuous flow synthesis revealed significant level of antibacterial activity against *Staphylococcus aureus* ATCC 43300 and *Escherichia coli* ATCC 12435 bacterial strains. The quantitative antimicrobial tests conducted by Stanića et al. [49] showed that the zinc and copper doped HAp exhibit viable cells reduction of *E. coli* ATCC 25922, *S. aureus* ATCC 25923 and antimicrobial activity was similar for samples doped with zinc and copper. The zinc and copper doped HAp samples analyzed by Stanića et al. were prepared by modified neutralization method in an inert atmosphere (N_2_). On the other hand, Mocanu et al. [50] showed that the zinc doped hydroxyapatite obtained by wet chemical approach without silver nanoparticles did not show any antibacterial effect against *E. coli* ATCC 10536, *S. aureus* ATCC 6583 P and *Staphylococcus* spp. (gangrenous mastitis) bacterial strains. Kim et al. [51], in their studies on antimicrobial effects of metal ions (Ag^+^, Cu^2+^, and Zn^2+)^ in hydroxyapatite also prepared by a wet chemical process, mentioned that the bactericidal effect against *E. coli* is difficult to ascertain when the hydroxyapatite was doped with copper and zinc.

Furthermore, Nzengue et al. [52] have shown in their studies that zinc plays a physiological role in major metabolic pathways. Moreover, Prasad et al. [53] showed that zinc is involved in growth and cell proliferation. On the other hand, an excessive concentration of zinc may have a neurotoxic effect leading to inhibition of enzymatic activities [54]. In addition, based on studies found in literature [55,56], in our research we also investigated the HepG2 cell viability in the presence of Zn:HAp and the antibacterial assay of Zn:HAp on *S. aureus* and *E. coli* bacterial strains. On the other hand, no significant differences of the cytotoxic effects were observed in the case of HepG2 cells. Previous studies conducted by Ito et al. [57] showed that the cytocompatibility of Zn^2+^ is often described as a non-toxic level. Nevertheless, some previous studies [58,59] showed that the substitution of Ca^2+^ with divalent or trivalent ions must be kept at a very low limit due to their toxicity. 

The physico-chemical analysis showed that the Zn^2+^ ions were incorporated in the hydroxyapatite structure by substituting Ca^2+^ ions. The (Ca+Zn)/P molar ratios determined from EDX and XPS analyzes are only slightly different. A lower value of (Ca+Zn)/P ratio obtained from XPS study may suggest that the (Ca+Zn)/P ratio is lower at the surface of Zn:HAp particles than within their bulk [60]. In agreement with previous studies presented by Tsuchida et al. [60], this behavior might suggest that HAp’s calcium and zinc ions, considered related to basic properties, are not readily expressed at the surface level. On the other hand, the values obtained for the samples analyzed are consistent with studies on surface characterization of hydroxyapatite presented by H. B. Lu et al. [61]. They showed that when the composition of the surface represents 1%–10% of the bulk ones, the powders of analyzed samples have surface stoichiometries similar to their bulk crystal compositions [61].

The increase of zinc concentration in the samples produced a broadening of the diffraction peaks and decrease of their intensities. This behavior was also observed in the infrared spectra. The broadening of infrared bands and the decrease of their intensities occurred due to zinc incorporation in the hydroxyapatite structure. This behavior is especially noticeable for the band found at around 632 cm^−1^ which is attributed to the hydroxyl librational (L(OH)) mode, and for the band from 3572 cm^−1^ which is attributed to the hydroxyl stretching mode. It is known that the bands from 632 cm^−1^ and 3572 cm^−1^ characterize the O–H vibrations in pure HAp. According to previous studies conducted by Guerra-López et al. [62] on the influence of nickel on hydroxyapatite crystallization, a progressive decrease in the intensity and a slight displacement of the position of the bands from 632 cm^−1^ and 3572 cm^−1^ to smaller wavelengths can be observed when the metal concentration substituted in the solid phase increases. On the other hand, the decrease of the infrared bands intensities when the zinc concentration increase, showed that the zinc was incorporated in the hydroxyapatite structure and induced a distortion in the phosphate environment due to smaller radius of zinc cation [63]. The XRD and FTIR results presented in this work showed that when low concentration of zinc were incorporated into the hydroxyapatite, the final product (Zn:HAp) exhibited a structural similarity to hexagonal HAp. On the other hand, previous studies have shown that the crystallinity decreased and the water content augmented when the zinc concentration in the HAp increased [62,63,64,65].

The studies conducted in recent years revealed that toxicity of nanoparticles of the same or different size and shape showed a toxic response varied. In the recent research on cytotoxicity of nanoparticles conducted by Lewinski et al. [66] showed that the toxicity of quantum dots to silica nanotubes are dependent of the size. During the same period, Pan et al. [67] were demonstrated the size-dependent cytotoxicity of gold nanoparticles. More recently, Zhang et al. [68] noted that different nanoparticle with same size exhibited differential toxicity on human fetal lung fibroblast. On the other hand, a differential toxicity of nanoparticles on primary mouse embryo fibroblast was reported by Yang et al. [69]. Moreover, Yang et al. [69] also showed that besides the size, shape and surface area of nanoparticles an important role is played and the type of cells exposed to nanoparticle. The findings reported so far had different results and conflicting regarding the toxicity of nanoparticles, which leads to the necessity to realize comprehensive and well-structured investigation depending on the size, shape, and surface area of nanoparticles and also of the type of cells.

The results of our studies bring new contributions on the biological properties such as antimicrobial activity and cell viability of Zn:HAp biocomposites. It was shown that the antimicrobial properties of HAp are influenced by many factors, such as stability of the colloidal dispersions, zinc concentration, synthesis method and not least the type of microbial strain tested. Moreover, in our opinion, further analysis of metal ions (Ag^+^, Cu^2+^, and Zn^2+^) in hydroxyapatite nanoparticles is needed to assess the antimicrobial properties and cell viability depending on stability of colloidal dispersions of metal ions concentration in the samples. For a correct assessment of the appropriate amount of Zn^2+^ ions that can be safely incorporated into the hydroxyapatite structure by substituting Ca^2+^ ions, other characterizations, as well as in vivo and in vitro experiments, should be conducted.

## 3. Materials and Methods

### 3.1. Sample Preparation

#### 3.1.1. Materials

In order to synthesize the zinc doped hydroxyapatite (Zn:HAp), precursors of calcium nitrate [Ca(NO_3_)_2_∙4H_2_O, Aldrich, Carlsbad, CA, USA), ammonium hydrogen phosphate ((NH_4_)2HPO_4_; Wako Pure Chemical Industries Ltd. (Richmond, VA, USA) and Zn(NO_3_)_6_·6H_2_O (Alpha Aesare, Karlsruhe, Germany, 99.99% purity) were used.

#### 3.1.2. Zinc Doped Hydroxyapatite (Zn:HAp) Nanoparticles

Zn doped hydroxyapatite (Zn:HAp) was synthetized by a modified co-precipitation method [15]. Zn:HAp, Ca_10−*x*_Zn*_x_*(PO_4_)_6_(OH)_2_ with 0.01 ≤ *x*_Zn_ ≤ 0.05 were synthesized under atmospheric pressure at a temperature of 80 °C using aqueous solutions with various Zn:(Zn+Ca) ion ratios, the [Ca+Zn]/P ratio being 1.67 [15,16]. Appropriate amounts of Ca(NO_3_)_2_·4H_2_O and Zn(NO_3_)_2_·6H_2_O were dissolved in deionized water. The pH value of the suspension was adjusted to 10 by adding 28% ammonia solution. A phosphate solution was prepared by dissolving (NH_4_)_2_HPO_4_ into deionized water. The (NH_4_)_2_HPO_4_ solution was added to the Ca(NO_3_)_2_·4H_2_O/Zn(NO_3_)_2_·6H_2_O solution at room temperature under constant stirring for 72 h at 80 °C. The resulting precipitate was filtered, washed five times with deionized water and centrifuged. Finally, the obtained Zn:HAp precipitates were dried in an oven at 80 °C for 72 h.

### 3.2. Characterization Methods

The structural characteristics of Ca_10−*x*_Zn*_x_*(PO_4_)_6_(OH)_2_ with 0.01 ≤ *x*_Zn_ ≤ 0.05 were investigated by X-ray diffraction (XRD), Fourier Transform-Infrared spectroscopy (FTIR) and X-ray photoelectron spectroscopy (XPS) analyses. The crystal phase was determined by powder XRD) measurements using a Bruker D8 Advance diffractometer (Bruker, Karlsruhe, Germany), with nickel filtered Cu_K__α_ (λ = 1.5418 Å) radiation and a high efficiency one-dimensional detector (Lynx Eye type) operated in integration mode. The samples were scanned in the 2θ range 20°–80°, with a step of 0.02° and 34 s measuring time per step. The secondary device was useful for the identification of the elemental composition. In order to highlight the functional groups, present in the prepared samples, FTIR spectra have been acquired using a Spectrum BX Spectrometer (PerkinElmer, Waltham, MA, USA). For these measurements, 1% of the powder was mixed and ground with 99% KBr. The powder mixture was then pressed at a load of 5 tons for 2 min obtaining pellets of 10 mm diameter. The spectrum was recorded in the range of 400 to 4000 cm^−1^ with a resolution of 4 and 128 times scanning. XPS measurements were carried out using a SPECS Multimethod Surface Analysis System (SPECS GmbH, Berlin, Germany) using monochromatic Al_Kα_ radiation (1486.6 eV). The vacuum in the analyzer chamber was p ~ 3 × 10^−9^ Torr. The X-rays are emitted by an anti-cathode of Al, U = 12.5 kV, with filament emission current I = 20 mA. For charge compensation, a FG40 flood gun had been used, providing an electron beam of 2 eV and 0.3 mA. The XPS recorded spectrum involved an energy window w = 20 eV with the resolution R = 20 eV, and with 400 recording channels. The XPS spectra were processed using Spectral Data Processor v2.3 (SDP) software. The colloidal properties of the Ca_10−*x*_Zn*_x_*(PO_4_)_6_(OH)_2_ with 0.01≤ *x*_Zn_ ≤0.05 nanocomposites were investigated by Dynamic Light Scattering (DLS) and zeta potential using dynamic light scattering (SZ-100 Nanoparticle Analyzer, HORIBA, Ltd., Kyoto, Japan) at 25 ± 1 °C. All the samples were diluted in distilled water before analysis. Before measuring of zeta potential, all samples were sonicated for 15 min. The pH of Zn:HAp suspension was equal to 7. For each sample analyzed, three determinations were recorded. The final value was determined by averaging the three measurements. The textural characteristics of synthesized nanocomposites were investigated by low temperature N_2_ adsorption desorption using a Micromeritics ASAP 2020 Physisorption Analyzer (Micromeritics Instrument Corp., Norcross, GA, USA) Scanning electron microscopy (SEM) has been used to show the morphology of all the samples using a HITACHI S2600N-type microscope (Hitachi High Technologies America, Inc., Schaumburg, IL, USA) equipped with an energy dispersive X-ray attachment (EDAX/2001 device).

### 3.3. Cytotoxicity Assays

The various Zn:HAp compounds were solubilized in water to a final concentration of 10 mg/mL.

#### 3.3.1. Antimicrobial Assays on Staphylococcus Aureus and Escherichia Coli Strain

The quantitative assay of the minimal inhibitory concentration (MIC, μg/mL) was based on two-fold serial dilutions performed in 96 well plates. For this purpose, serial dilutions of the tested compounds (ranging between 1000 and 1.95 μg/mL) were performed final volumes of 200 μL in Luria Bertani medium. Each well was inoculated with the bacterial (*S. aureus* ATCC 25923 and *E. coli* MG1655) strain at a 600 nm absorbance of 0.02. The plates were incubated for 12 h at 30 °C with constant shaking at 45 rpm in a Tecan plate reader and the absorbance at 600 nm was measured every 15 min.

#### 3.3.2. HepG2 Cell Viability Assays

HepG2 cell growth assays were performed as previously described [17] with the following modifications: HepG2 cells were grown in Minimum Essential Medium (MEM) supplemented with 10% *v*/*v* fetal bovine serum (FBS), 20 mM L-glutamine, 100 μg·mL^−1^ streptomycin and 100 U·mL^−1^ penicillin. Cells were cultured at 37 °C in a humidified atmosphere with 5% CO_2_. The culture medium was renewed every 2 to 3 days.

For cell viability assays, 3.105 HepG2 cells were seeded in each well of a 12-well plates. On the following day, Zn:HAp were added at 0, 62.5, 125, 250 and 500 µg/mL for 24 h. Cells were rinsed twice with PBS, harvested using trypsin and suspended in 200 µL PBS. Cell viability was determined by counting Trypan Blue stained cells in a TC20 Automated Cell Counter (Bio-Rad, Hercules, CA, USA).

## 4. Conclusions

The current study investigated the structural and biological properties of hydroxyapatite doped with low concentrations of zinc (0.01< *x*_Zn_ < 0.05). The results presented in this study indicate that the Zn^2+^ ions are incorporated into the hydroxyapatite lattice and the structure of the final Zn:HAp is similar to hexagonal HAp. The XRD investigations showed the formation of a hexagonal phase with lattice parameters that are characteristic to hydroxyapatite. The incorporation of Zn^2+^ ions in the HAp structure conducted to a decrease of the crystallinity of the samples when the zinc concentration increased. The FTIR analysis revealed that the characteristic bands are assigned to vibrational modes of phosphate, carbonate and hydroxyl groups. On the other hand, the FTIR analysis showed that the water content increased when the zinc concentration increased. Moreover, SEM and XRD investigations showed a decrease of the particle size when the concentration of zinc increased.

The cytotoxic effect of HAp on prokaryote and eukaryote cells was also investigated. We have demonstrated that the stability of the colloidal dispersions, zinc concentration, and synthesis method played an important role on cell viability. Besides, we have proven that the antimicrobial properties of HAp were also dependent on the type of microbial strain. We conclude that the Zn:HAp nanoparticles have the potential to be used in biomedical field. Our future work will be focused on studies for a better understanding of interaction between ZnHAp nanoparticles and cell membranes of Gram-negative and Gram-positive bacteria depending on the stability of the dispersions, zinc concentration and size.

## Figures and Tables

**Table 1:** Calculated lattice constants, the deviation of Zn:HAp and the average crystallite sizes measured parallel and perpendicular to the c axis with different *x*_Zn_ values.

Sample	Cell Parameters		Crystallite Sizes (Å)
	a = b/Deviation (Å)	c/Deviation (Å)	
ICDD-PDF#9-432	9.418	6.884	-
*x*_Zn_ = 0	9.4320/0.001	6.8838/0.001	231.879 ± 1.000
*x*_zn_ = 0.01	9.4325/0.001	6.8828/0.001	230.797 ± 1.000
*x*_zn_ = 0.03	9.4348/0.001	6.8818/0.001	224.182 ± 1.000
*x*_zn_ = 0.04	9.4365/0.001	6.8808/0.002	201.651 ± 1.000

**Table 2:** Summary of N_2_ adsorption/desorption results.

Sample	BET Nitrogen Adsorption/Desorption			BJH Nitrogen Adsorption/Desorption	
	S_BET_ (m^2^/g)	V_m_ (cm^3^)	C	Pore Size (nm)	Pore Volume (m^3^/g)
*x*_Zn_ = 0.01	99.481	22.852	386.660	18.533	0.461
*x*_Zn_ = 0.03	104.285	23.956	383.430	15.485	0.404
*x*_Zn_ = 0.05	115.421	26.514	393.876	12.408	0.358
